# Performance Comparison between Fountain Codes-Based Secure MIMO Protocols with and without Using Non-Orthogonal Multiple Access

**DOI:** 10.3390/e21100982

**Published:** 2019-10-09

**Authors:** Dang The Hung, Tran Trung Duy, Phuong T. Tran, Do Quoc Trinh, Tan Hanh

**Affiliations:** 1 Faculty of Radio-Electronics Engineering, Le Quy Don Technical University, Ha Noi 100000, Vietnam; danghung8384@gmail.com (D.T.H.); trinhdq@mta.edu.vn (D.Q.T.); 2 Department of Telecommunications, and Department of Information Technology, Posts and Telecommunications Institute of Technology, Ho Chi Minh City 700000, Vietnam; trantrungduy@ptithcm.edu.vn (T.T.D.); tanhanh@ptithcm.edu.vn (T.H.); 3 Wireless Communications Research Group, Faculty of Electrical and Electronics Engineering, Ton Duc Thang University, Ho Chi Minh City 700000, Vietnam

**Keywords:** physical-layer security, fountain codes, non-orthogonal multiple access, intercept probability

## Abstract

In this paper, we propose and evaluate the performance of fountain codes (FCs) based secure transmission protocols in multiple-input-multiple-output (MIMO) wireless systems, in presence of a passive eavesdropper. In the proposed protocols, a source selects its best antenna to transmit fountain encoded packets to a destination that employs selection combining (SC) or maximal ratio combing (MRC) to enhance reliability of the decoding. The transmission is terminated when the destination has a required number of the encoded packets to reconstruct the original data of the source. Similarly, the eavesdropper also has the ability to recover the source data if it can intercept a sufficient number of the encoded packets. To reduce the number of time slots used, the source can employ non-orthogonal multiple access (NOMA) to send two encoded packets to the destination at each time slot. For performance analysis, exact formulas of average number of time slots (TS) and intercept probability (IP) over Rayleigh fading channel are derived and then verified by Monte-Carlo simulations. The results presented that the protocol using NOMA not only reduces TS but also obtains lower IP at medium and high transmit signal-to-noise ratios (SNRs), as compared with the corresponding protocol without using NOMA.

## 1. Introduction

Secure communication is one of the critical issues of wireless communication systems due to the broadcast nature of wireless channels. Conventionally, cryptographic methods at upper layers are used to obtain wireless security via generating cryptographic keys. However, eavesdroppers can decode the encrypted signals if they are equipped with advanced equipment and have enough time for the decoding operation. In [1,2,3,4,5,6], the authors introduced a new security method, called physical-layer security (PLS), where characteristics of wireless channels, i.e., distances and channel state information (CSI), can be exploited to ensure confidentiality of the data transmission. To obtain the security in PLS, the secrecy capacity must be greater than zero or the channel capacity of the data link must be better than that of the eavesdropping link. For example, joint transmit and receive diversity methods [7,8,9,10] were proposed to enhance secrecy performances for multiple-input-multiple-output (MIMO) secure communication protocols, in terms of secrecy outage probability (SOP) and probability of non-zero secrecy capacity (PNSC). Particularly, the transmitters in [7,8,9,10] select the best transmit antenna (transmit antenna selection (TAS)) to maximize post-processed signal-to-noise ratios (SNRs) obtained at the intended receivers that use maximal ratio combining (MRC) or selection combining (SC). Because the eavesdroppers in [7,8,9,10] only obtain the receive diversity with their MRC or SC combiners, the diversity order of the data links can be higher than that of the eavesdropping ones. In [11], the secrecy outage performance of the TAS/MRC method in underlay cognitive radio networks (CRNs) was evaluated. In the underlay spectrum sharing approach, transmit power of the secondary transmitters is limited by a pre-determined interference level so that quality of service (QoS) of the primary network is not harmful. In contrast to [11], the authors in [12] proposed a secure transmission protocol in overlay CRNs. In this system model, a full-duplex secondary transmitter employs TAS/MRC to transmit the secondary data and receive the primary data at the same time. Moreover, it can use an interactive zero forcing beam-forming method to simultaneously broadcast both the primary and secondary data. The protocol proposed in [12] not only enhances the SOP performance for the primary network but also improves throughput of the secondary transmission. Published works [13,14] introduced the PLS schemes in radio frequency energy harvesting (RF-EH) environment. In [13], one multi-antenna base station adopts TAS to send information and energy to one desired receiver and EH receivers, respectively. Since the EH receivers can illegally decode the information of the intended receiver, there exists a trade-off between energy harvested and security of the data transmission. In [14], an energy-limited source harvests the RF energy from a dedicated power beacon for transmitting the data in presence of multiple eavesdroppers. In addition, the source can employ TAS or maximal ratio transmission (MRT) to enhance the secrecy diversity order. Recently, secure transmission approaches for non-orthogonal multiple access (NOMA) systems have been studied. In contrast to conditional transmission techniques, the source using NOMA can send multiple signals to the destinations at the same time, frequency and code. Indeed, the signals that are linearly combined with different transmit power levels are then sent to the destinations which use successive interference cancellation (SIC) to extract the desired signals. In [15], the authors proposed various TAS methods to enhance the secrecy performance for two-user down-link NOMA networks. Reference [16] investigated the SOP performance of a secure NOMA system using max-min TAS method, in presence of non-colluding and colluding eavesdroppers.

Cooperative relaying protocols with efficient relay selection methods [17,18,19] also provide high secrecy performance for PLS-based wireless networks. The advantages of these schemes are that (i) the data transmission on short hops is more reliable, (ii) the relay selection provides high diversity gain. However, because the source data can be overheard over multiple hops, the channel capacity obtained at the eavesdroppers can be significantly increased by using the MRC combiner [20]. To solve this problem, a randomize-and-forward strategy [20,21] is often employed by the transmitters including the source and the relays to confuse the eavesdroppers. In [22], a secure transmission protocol in a dual-hop MIMO relay system using TAS/MRC over Nakagami-*m* fading channels was proposed and analyzed. The authors of [23] considered a buffer-aided MIMO cooperative system in the presence of a passive eavesdropper. Particularly, due to lack of the CSI of the eavesdropping channel, a joint transmit antenna and relay selection scheme was proposed to only enhance the quality of the main channel. Published works [24,25] analyzed SOP of dual-hop cooperative underlay CRNs with and without direct link between the secondary source and the secondary destination. In [26], secure communication protocols in multi-hop underlay CRNs were considered. In addition, the authors in [26] introduced an efficient cooperative routing method to enhance the end-to-end secrecy performance, as compared with the traditional mutli-hop transmission one. To further enhance the secrecy performance for cooperative cognitive networks, cooperative jamming (CJ) [27,28] can be used. With CJ, jammers are employed to transmit interference on the eavesdroppers, while the intended receivers can remove the interference from their received signals via cooperation with jammers. However, the implementation of the CJ methods is very complex due to a high synchronization between the jammer and receiver nodes. Moreover, the jamming signals can cause co-channel interference on other wireless devices in the network. In [29], the authors proposed a secure two-way relaying protocol, where two legitimate users exchange data with each other via the help of amplify-and-forward cooperative relays, with presence of an eavesdropper, and imperfect CSI of the eavesdropping channels. References [30,31,32] considered secure transmission protocols in RF-EH relay systems, in which the relay nodes have to harvest energy from the RF signals to forward the source data to the destination. In [32], the destination plays a role as a jammer for obtaining positive secrecy rate with presence of the untrusted relay. In [33,34,35], wireless powered CJ methods are employed to improve the secrecy rate. In these methods, called harvest-to-jam (HJ), the jammer nodes first harvest energy from ambient RF sources and then use the harvested energy to generate noises. References [36,37] investigated the secrecy performance of cooperative NOMA systems with various relay selection methods. In [38], the source performs the jamming operation to enhance the security for dual-hop relaying networks using NOMA. In [39,40], secure NOMA transmission strategies in CRNs were proposed and analyzed. In [41], the trade-off between security and reliability of cooperative cognitive NOMA systems was evaluated via SOP and connection outage probability (COP).

Fountain codes (FCs) or rateless codes [42,43] have gained much attention due to low decoding complexity. In contrast to typical fixed-rate codes, a FC transmitter can generate a limitless stream of fountain encoded packets from a finite number of the source packets. The encoded packets are then continuously sent to the desired receivers until each receiver can receive a sufficient number of the encoded packets for recovering the original data (regardless of which encoded packets are received). Therefore, FCs do not require knowledge of CSI, automatically adapt the channel conditions, and avoid the feedback channel. In [44], the authors proposed a FCs based cooperative relaying network, where energy consumption and transmission time significantly decrease due to mutual information accumulation. Published work [45] presented the advantage of applying FCs on wireless broadcast systems, in terms of transmission efficiency. In [46], a rateless code based spectrum access model in overlay CRNs was proposed. In the scheme proposed in [46], the secondary transmitters help a primary transmitter forward the fountain packets to a primary receiver, and then they can find opportunities to access licensed bands. The authors of [47] considered cooperative relay networks using FCs and RF-EH, where the source and relay nodes use FCs, and hence, the destination can perform the mutual information accumulation and energy accumulation. However, due to broadcast of wireless channels, the eavesdroppers can also receive enough number of the encoded packets for intercepting the original data. Hence, security in FCs based PLS system becomes a critical issue.

### 1.1. Related Work

Until now, there have been many published works concerned with performance analysis of diversity based secure communication using MIMO techniques, e.g., [7,8,9,10,11,12,13,14,15,16], and cooperative relaying methods [17,18,19,20,21,22,23,24,25,26,27,28,29,30,31,32,33,34,35,36,37,38,39,40,41]. However, to the best of our knowledge, several existing literatures studying secure transmission protocols using FCs have been reported. The basic idea of the FC-based PLS protocols is that when the intended destination can receive enough encoded packets before the eavesdroppers, the data transmission is successful and secure [48]. In [49], the authors evaluated the intercept probability which is defined as the probability that the eavesdropper can intercept enough coded packets to recover the original data. In [50], the authors proposed a multicast model to attain the wireless security for Internet of Things (IoT) networks using FCs. In [51], the secrecy performance of the FCs aided PLS protocol is significantly enhanced with the TAS and CJ techniques when the transceiver hardware of the destination and the eavesdropper are not perfect. Reference [52] considered a FCs aided relaying network using the CJ method to enhance the transmission secrecy, in terms of quality-of-service violating probability (QVP). In [53], the authors proposed various relay selection and jammer selection methods to enhance both outage performance and IP performance for dual-hop multiple-relay decode-and-forward networks. The authors of [54] proposed a FCs based transmission protocol to secure the source-destination communication. Moreover, a new FC construction method, which opportunistically adapts the coding strategy following outage prediction, is proposed in [54]. In [55], the authors analyzed the security-reliability trade-off for multi-hop low-energy adaptive clustering hierarchy (LEACH) networks employing FCs and CJ. The authors of [56] proposed a rateless codes-based communication protocol to provide security for wireless systems. In this protocol, a source uses the TAS technique to transmit the encoded packets to a destination, and a cooperative jammer harvests energy from the RF signals of the source and interference sources to generate jamming noises on an eavesdropper.

### 1.2. Motivations and Contributions

In this paper, we propose a MIMO secure communication system exploiting FCs. In the proposed protocol, a multi-antenna source uses TAS to transmit the encoded packets to a multi-antenna destination in presence of a multi-antenna eavesdropper. The receivers including the destination and the eavesdropper can use the MRC or SC combiner to enhance the reliability of the decoding operation. When a required number of the encoded packets can be obtained by the destination, it sends a feedback to the source for stopping the transmission. Therefore, the security is guaranteed as the eavesdropper cannot sufficiently intercept the encoded packets. The main motivations and contributions of this paper can be summarized as follows:

- In contrast to [48,49,50,54], in the proposed protocol, all the nodes including the source, the destination and eavesdropper are equipped with multiple antennas and use the MRC or SC technique to combine the received signals. Although the source nodes in [51,56] have multi-antenna and employ TAS to transmit the encoded packets, the destinations in [51,56] are only single-antenna nodes. Moreover, References [52,53,55] considered single-input-single-output (SISO) relaying protocols where all the terminals are deployed with a single antenna.
- In contrast to [48,49,50,51,52,53,54,55,56], the source in the proposed protocol can employ NOMA to transmit two packets to the destination in each time slot to reduce the number of time slots used. Moreover, reducing the number of time slots also means reducing the delay time and transmit power, which are important metrics of the wireless systems.
- We compare the performance of the proposed protocols in two cases where the source uses NOMA (named NOMA) and does not use NOMA (named Wo-NOMA), in terms of average number of time slots (TS) and intercept probability (IP). The results shows that the FCs based secure transmission protocol exploiting NOMA can decrease both TS and IP, as compared with the corresponding protocol without using NOMA.
- We derive exact expressions of TS and IP for the NOMA and Wo-NOMA protocols over Rayleigh fading channels and realize computer simulations to verify.

The remainder of this paper is organized as follows. The system model of NOMA and Wo-NOMA is described in Section 2. In Section 3, the TS and IP performances of NOMA and Wo-NOMA over Rayleigh fading channel are evaluated. The simulation and theoretical results are shown in Section 4. Finally, this paper is concluded in Section 5.

## 2. System Model

**Figure 1:**
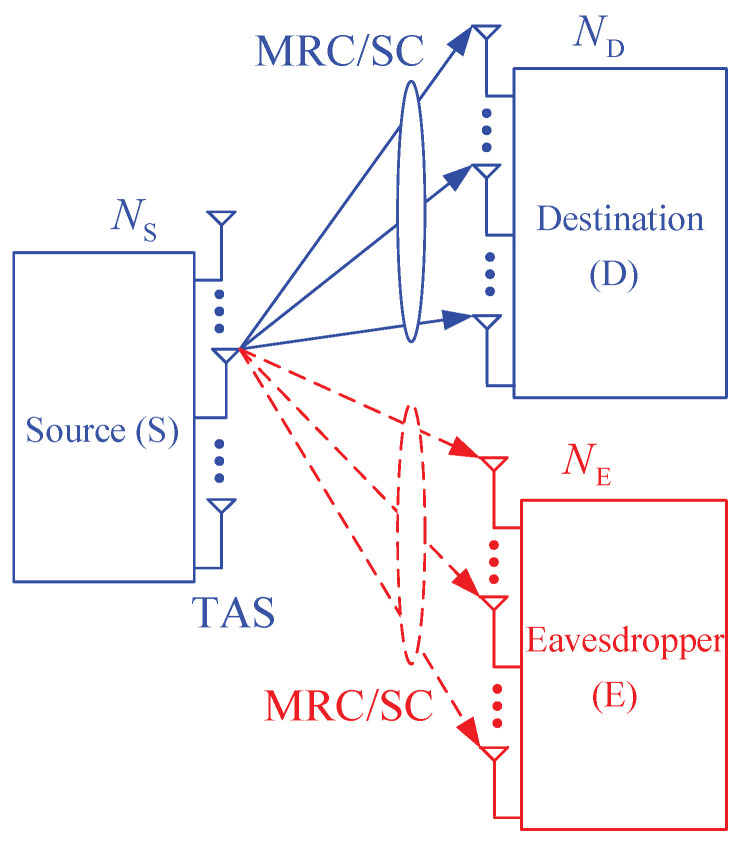
System model of the proposed scheme.

Figure 1 presents system model of the proposed protocol, where a source node (S) equipped with $N_{S}$ antennas uses FCs to transmit its data to an $N_{D}$-antenna destination (D), in presence of an $N_{E}$-antenna passive eavesdropper (E). The original data of the source is divided into *L* packets which are then encoded by the FC encoder. At each time slot, the source selects its best antenna to transmit two (or one) encoded packets to the destination, which are also received by the eavesdropper. Then, the D and E nodes attempt to decode the encoded packets. To recover the original data, the destination and eavesdropper have to correctly receive at least $N_{\mathrm{req}}^{\mathrm{pkt}}$ encoded packets, where $N_{\mathrm{req}}^{\mathrm{pkt}}=\left(1+\varepsilon \right)L$, and ε is the decoding overhead which depends on concrete code design [48,49,50,51,52,53,54,55,56]. After receiving a sufficient number of the encoded packets for reconstructing the original data, the destination sends an ACK message to inform the source, and then the source stops its transmission. In this case, if the eavesdropper successfully receives at least $N_{\mathrm{req}}^{\mathrm{pkt}}$ encoded packets, it can also recover the original data, and hence the source data is intercepted.

Next, we introduce notations and assumptions used through this paper. Let us denote $h_{S_{m}D_{n}}$ and $h_{S_{m}E_{t}}$ as channel coefficients between the *m*-th antenna of the source and *n*-th antenna of the destination and between the *m*-th antenna of the source and *t*-th antenna of the eavesdropper, respectively, where $m=1,2,\ldots ,N_{S}$, $n=1,2,\ldots ,N_{D}$, $t=1,2,\ldots ,N_{E}$. We assume that all the channels are independent and identically distributed (i.i.d.), block and flat Rayleigh fading, where they keep constant in one time slot but independently changes at other time slots. Therefore, the channel gains $\gamma_{S_{m}D_{n}}=|h_{S_{m}D_{n}}{|}^{2}$ and $\gamma_{S_{m}E_{t}}=|h_{S_{m}E_{t}}{|}^{2}$ are exponential random variables (RVs) whose cumulative distribution functions (CDFs) are expressed respectively as [57]:

(1) $$\begin{array}{cc} F_{\gamma_{S_{m}D_{n}}}\left(x\right) & =1-\exp\left(-\lambda_{\mathrm{SD}}x\right),\\ F_{\gamma_{S_{m}E_{t}}}\left(x\right) & =1-\exp\left(-\lambda_{\mathrm{SE}}x\right), \end{array}$$

where $\lambda_{\mathrm{SD}}=1/E\left\{\gamma_{S_{m}D_{n}}\right\}$ and $\lambda_{\mathrm{SE}}=1/E\left\{\gamma_{S_{m}E_{t}}\right\}$, and $E\left\{.\right\}$ is an expected operator.

Therefore, probability density function (PDF) of $\gamma_{S_{m}D_{n}}$ and $\gamma_{S_{m}E_{t}}$ can be given respectively as

(2) $$\begin{array}{cc} f_{\gamma_{S_{m}D_{n}}}\left(x\right) & =\lambda_{\mathrm{SD}}\exp\left(-\lambda_{\mathrm{SD}}x\right),\\ f_{\gamma_{S_{m}E_{t}}}\left(x\right) & =\lambda_{\mathrm{SE}}\exp\left(-\lambda_{\mathrm{SE}}x\right). \end{array}$$

Let $N_{\mathrm{TS}}$ denote number of time slots used by the source to transmit the encoded packets to the destination. We denote $N_{D}^{\mathrm{pkt}}$ and $N_{E}^{\mathrm{pkt}}$ as number of the encoded packets that the destination and the eavesdropper can successfully receive, respectively.

Function $\left\lfloor x\right\rfloor$ gives the greatest integer less than or equal to *x*, and function $\left\lceil x\right\rceil$ gives the smallest integer equal to or greater than *x*.

### 2.1. Without Using NOMA (Wo-NOMA)

If the source does not use NOMA, at each time slot, it transmits one encoded packet to the destination. Assume that each encoded packet, e.g., *p*, includes *U* symbols, i.e., $p=\left\{x_{1}\left[1\right],x_{1}\left[2\right],\ldots ,x_{1}\left[U\right]\right\}$, where $x\left[u\right]$ is a symbol of *p*, and u=1,2,…,U. When the source uses the *m*-th antenna to transmit $x_{u}$ to the destination, the received signal at the *n*-th antenna of the destination is expressed as

(3) $$\begin{array}{c} y_{D}\left[u\right]=\sqrt{P}h_{S_{m}D_{n}}x\left[u\right]+n_{D}\left[u\right], \end{array}$$

where *P* is transmit power of all the antennas of the source, $n_{D}\left[u\right]$ is additive white Gaussian noise (AWGN) at D. For ease of presentation and analysis, we assume that all the additive noises are modeled as Gaussian RVs with zero mean and variance of $\sigma^{2}$.

From (3), the instantaneous signal-to-noise ratio (SNR) of the $S_{m}\to D_{n}$ link is given as

(4) $$\begin{array}{c} \psi_{S_{m}D_{n}}=\frac{P\gamma_{S_{m}D_{n}}}{\sigma^{2}}=\Delta \gamma_{S_{m}D_{n}}, \end{array}$$

where $\Delta =P/\sigma^{2}$ is transmit SNR.

When the destination uses the SC technique, the SNR obtained at the output of the combiner can be formulated similarly to Equation (3) of [58] as

(5) $$\begin{array}{c} \psi_{S_{m}D_{b}}=\max_{n=1,2,\ldots ,N_{D}}\left(\psi_{S_{m}D_{n}}\right), \end{array}$$

where *b* denotes index of the receive antenna at D used to decode $x\left[u\right]$, $b\in \left\{1,2,\ldots ,N_{D}\right\}$.

Then, the source selects its best antenna to maximize the instantaneous SNR of the data link (see [51]):

(6) $$\begin{array}{c} \psi_{S_{a}D_{b}}=\max_{m=1,2,\ldots ,N_{S}}\left(\psi_{S_{m}D_{b}}\right), \end{array}$$

where *a* denotes index of the selected transmit antenna at the source.

Combining (5) and (6), we can rewrite the SNR of the data link as

(7) $$\begin{array}{c} \psi_{D}^{\mathrm{TAS}/\mathrm{SC}}=\max_{m=1,2,\ldots ,N_{S}}\left(\max_{n=1,2,\ldots ,N_{D}}\left(\psi_{S_{m}D_{n}}\right)\right). \end{array}$$

For a fair comparison, the eavesdropper also uses the SC combiner for decoding *p*. Similar to (5), the obtained SNR of the eavesdropping link is computed as

(8) $$\begin{array}{c} \psi_{E}^{\mathrm{SC}}=\max_{t=1,2,\ldots ,N_{E}}\left(\psi_{S_{a}E_{t}}\right), \end{array}$$

where $\psi_{S_{a}E_{t}}=\Delta \gamma_{S_{a}E_{t}}$.

If the destination uses MRC, the combined signal at D can be given as

(9) $$\begin{array}{cc} y_{D}^{\mathrm{MRC}}\left[u\right] & =\sum_{n=1}^{N_{D}}\frac{\sqrt{P}h_{S_{m}D_{n}}^{*}}{\sum_{n=1}^{N_{D}}P|h_{S_{m}D_{n}}{|}^{2}}y_{D}\left[u\right]\\ \; & =x\left[u\right]+\sum_{n=1}^{N_{D}}\frac{\sqrt{P}h_{S_{m}D_{n}}^{*}n_{D}\left[u\right]}{\sum_{n=1}^{N_{D}}P|h_{S_{m}D_{n}}{|}^{2}}, \end{array}$$

where $h_{S_{m}D_{n}}^{*}$ is conjugate of the complex number $h_{S_{m}D_{n}}$.

From (9), the SNR obtained at D is calculated as

(10) $$\begin{array}{c} \psi_{S_{m}D}^{\mathrm{MRC}}=\sum_{n=1}^{N_{D}}\Delta |h_{S_{m}D_{n}}{|}^{2}=\sum_{n=1}^{N_{D}}\psi_{S_{m}D_{n}}. \end{array}$$

Then, the TAS technique is employed to provide the highest SNR for the data link, i.e.,

(11) $$\begin{array}{c} \psi_{D}^{\mathrm{TAS}/\mathrm{MRC}}=\max_{m=1,2,\ldots ,N_{S}}\left(\sum_{n=1}^{N_{D}}\psi_{S_{m}D_{n}}\right). \end{array}$$

Similar to (10), the instantaneous SNR of the eavesdropping link is computed as

(12) $$\begin{array}{c} \psi_{E}^{\mathrm{MRC}}=\sum_{t=1}^{N_{E}}\psi_{S_{a}E_{t}}, \end{array}$$

where *a* denotes index of the selected antenna at the source.

**Remark** **1.** *Due to the block fading channel, the instantaneous SNRs of the symbols $x\left[u\right]$ are the same for all u. Hence, in *(7)*, *(8)*, *(11)* and *(12)*, we skip the index u as presenting SNRs of the data and eavesdropping channels. Next, we assume that the encoded packet p can be decoded successfully if the instantaneous SNRs received at the destination and the eavesdropper are higher than a predetermined threshold denoted by $\gamma_{\mathrm{th}}$, which can be formulated respectively as*

(13) $$\begin{array}{cc} \rho_{D} & =\mathrm{Pr}\left(\psi_{D}^{Y}\geq \gamma_{\mathrm{th}}\right),\\ \rho_{E} & =\mathrm{Pr}\left(\psi_{E}^{Z}\geq \gamma_{\mathrm{th}}\right), \end{array}$$

*where $Y\in \left\{\mathrm{TAS}/\mathrm{SC},\mathrm{TAS}/\mathrm{MRC}\right\}$ and $Z\in \left\{\mathrm{SC},\mathrm{MRC}\right\}$.*

Then, the probabilities that D and E nodes cannot correctly be decoded the encoded packet *p* are given as $1-\rho_{D}$ and $1-\rho_{E}$, respectively.

### 2.2. Using NOMA

To reduce the number of time slots used to transmit the encoded packets, the source can use NOMA to transmit two encoded packets, e.g., $p_{1}$ and $p_{2}$, to the destination in one time slot. We can assume that $p_{1}=\left\{x_{1}\left[1\right],x_{1}\left[2\right],\ldots ,x_{1}\left[U\right]\right\}$ and $p_{2}=\left\{x_{2}\left[1\right],x_{2}\left[2\right],\ldots ,x_{2}\left[U\right]\right\}$, where $x_{1}\left[u\right]$ and $x_{2}\left[u\right]$ are symbols of $p_{1}$ and $p_{2}$, respectively, and u=1,2,…,U. Indeed, the source linearly combines two signals $x_{1}\left[u\right]$ and $x_{2}\left[u\right]$ [36], i.e., $x_{+}\left[u\right]=\sqrt{a_{1}P}x_{1}\left[u\right]+\sqrt{a_{2}P}x_{2}\left[u\right]$, and it then sends $x_{+}\left[u\right]$ to the destination, where $a_{1}$ and $a_{2}$ are power allocation coefficients with $a_{1}+a_{2}=1$, $a_{1}>a_{2}>0$. Similar to (3), the received signal at D can be expressed as

(14) $$\begin{array}{cc} y_{D}\left[u\right] & =h_{S_{m}D_{n}}x_{+}\left[u\right]+n_{D}\left[u\right]\\ \; & =h_{S_{m}D_{n}}\left(\sqrt{a_{1}P}x_{1}\left[u\right]+\sqrt{a_{2}P}x_{2}\left[u\right]\right)+n_{D}\left[u\right]. \end{array}$$

Follows the SIC principle, the destination first decodes $x_{1}\left[u\right]$ by treating $x_{2}\left[u\right]$ as noise. After successfully decoding $x_{1}\left[u\right]$, D removes the component including $x_{1}\left[u\right]$, i.e., $\sqrt{a_{1}P}h_{S_{m}D_{n}}x_{1}\left[u\right]$, from $y_{D}\left[u\right]$. Then, the signal used to decode $x_{2}\left[u\right]$ can be expressed as (see [36])

(15) $$\begin{array}{c} z_{D}\left[u\right]=\sqrt{a_{2}P}h_{S_{m}D_{n}}x_{2}\left[u\right]+n_{D}\left[u\right]. \end{array}$$

From (14) and (15), the instantaneous SNRs, with respect to $x_{1}\left[u\right]$ and $x_{2}\left[u\right]$, are given respectively as

(16) $$\begin{array}{c} \psi_{S_{m}D_{n}}^{x_{1}\left[u\right]}=\frac{a_{1}\Delta \gamma_{S_{m}D_{n}}}{a_{2}\Delta \gamma_{S_{m}D_{n}}+1},\psi_{S_{m}D_{n}}^{x_{2}\left[u\right]}=a_{2}\Delta \gamma_{S_{m}D_{n}}. \end{array}$$

When the TAS/SC technique is employed, similar to (7), the obtained SNRs of the data link for decoding $x_{1}\left[u\right]$ and $x_{2}\left[u\right]$ can be expressed respectively as

(17) $$\begin{array}{cc} \psi_{D,1}^{\mathrm{TAS}/\mathrm{SC}} & =\frac{a_{1}\max_{m=1,2,\ldots ,N_{S}}\left(\max_{n=1,2,\ldots ,N_{D}}\left(\psi_{S_{m}D_{n}}\right)\right)}{a_{2}\max_{m=1,2,\ldots ,N_{S}}\left(\max_{n=1,2,\ldots ,N_{D}}\left(\psi_{S_{m}D_{n}}\right)\right)+1},\\ \psi_{D,2}^{\mathrm{TAS}/\mathrm{SC}} & =a_{2}\max_{m=1,2,\ldots ,N_{S}}\left(\max_{n=1,2,\ldots ,N_{D}}\left(\psi_{S_{m}D_{n}}\right)\right). \end{array}$$

Similarly, the eavesdropper E first decodes $x_{1}\left[u\right]$, and then performs SIC before decoding $x_{2}\left[u\right]$. With the SC combiner, the instantaneous SNRs of the eavesdropping channel used to decode $x_{1}\left[u\right]$ and $x_{2}\left[u\right]$ are given respectively as

(18) $$\begin{array}{c} \psi_{E,1}^{\mathrm{SC}}=\frac{a_{1}\max_{t=1,2,\ldots ,N_{E}}\left(\psi_{S_{a}E_{t}}\right)}{a_{2}\max_{t=1,2,\ldots ,N_{E}}\left(\psi_{S_{a}E_{t}}\right)+1},\psi_{E,2}^{\mathrm{SC}}=a_{2}\max_{t=1,2,\ldots ,N_{E}}\left(\psi_{S_{a}E_{t}}\right). \end{array}$$

In the case that the MRC technique is used, the combined signal at D can be given as

(19) $$\begin{array}{cc} y_{D,x_{1}}^{\mathrm{MRC}}\left[u\right] & =\sum_{n=1}^{N_{D}}\frac{\sqrt{a_{1}P}h_{S_{m}D_{n}}^{*}}{a_{1}P\sum_{n=1}^{N_{D}}|h_{S_{m}D_{n}}{|}^{2}}\left(\sqrt{a_{1}P}h_{S_{m}D_{n}}x_{1}\left[u\right]+\sqrt{a_{2}P}h_{S_{m}D_{n}}x_{2}\left[u\right]+n_{D}\left[u\right]\right)\\ \; & =x_{1}\left[u\right]+\frac{\sqrt{a_{2}}}{\sqrt{a_{1}}}x_{2}\left[u\right]+\sum_{n=1}^{N_{D}}\frac{\sqrt{a_{1}P}h_{S_{m}D_{n}}^{*}n_{D}\left[u\right]}{a_{1}P\sum_{n=1}^{N_{D}}|h_{S_{m}D_{n}}{|}^{2}}. \end{array}$$

After canceling the components including $x_{1}\left[u\right]$ from the signals received at all the antennas, the destination again uses MRC to decode $x_{2}\left[u\right]$ using the following combined signal:

(20) $$\begin{array}{cc} y_{D,x_{2}}^{\mathrm{MRC}}\left[u\right] & =\sum_{n=1}^{N_{D}}\frac{\sqrt{a_{2}P}h_{S_{m}D_{n}}^{*}}{a_{2}P\sum_{n=1}^{N_{D}}|h_{S_{m}D_{n}}{|}^{2}}\left(\sqrt{a_{2}P}h_{S_{m}D_{n}}x_{2}\left[u\right]+n_{D}\left[u\right]\right)\\ \; & =x_{2}\left[u\right]+\sum_{n=1}^{N_{D}}\frac{\sqrt{a_{2}P}h_{S_{m}D_{n}}^{*}n_{D}\left[u\right]}{a_{2}P\sum_{n=1}^{N_{D}}|h_{S_{m}D_{n}}{|}^{2}}. \end{array}$$

From (19) and (20), the obtained SNRs, with respect to $x_{1}\left[u\right]$ and $x_{2}\left[u\right]$, can be expressed respectively as

(21) $$\begin{array}{c} \psi_{S_{m}D}^{x_{1}\left[u\right]}=\frac{a_{1}\sum_{n=1}^{N_{D}}\psi_{S_{m}D_{n}}}{a_{2}\sum_{n=1}^{N_{D}}\psi_{S_{m}D_{n}}+1},\psi_{S_{m}D}^{x_{2}\left[u\right]}=a_{2}\sum_{n=1}^{N_{D}}\psi_{S_{m}D_{n}}. \end{array}$$

Since the source uses TAS to optimize quality of the data link, the obtained SNRs used to decode $x_{1}\left[u\right]$ and $x_{2}\left[u\right]$ can be calculated respectively as

(22) $$\begin{array}{cc} \psi_{D,1}^{\mathrm{TAS}/\mathrm{MRC}} & =\max_{m=1,2,\ldots ,N_{S}}\left(\frac{a_{1}\sum_{n=1}^{N_{D}}\psi_{S_{m}D_{n}}}{a_{2}\sum_{n=1}^{N_{D}}\psi_{S_{m}D_{n}}+1}\right),\\ \psi_{D,2}^{\mathrm{TAS}/\mathrm{MRC}} & =\max_{m=1,2,\ldots ,N_{S}}\left(a_{2}\sum_{n=1}^{N_{D}}\psi_{S_{m}D_{n}}\right). \end{array}$$

Similarly, for the eavesdropping channel, the instantaneous SNRs, with respect to $x_{1}\left[u\right]$ and $x_{2}\left[u\right]$, can be formulated respectively as

(23) $$\begin{array}{c} \psi_{E,1}^{\mathrm{MRC}}=\frac{a_{1}\sum_{t=1}^{N_{E}}\psi_{S_{a}E_{t}}}{a_{2}\sum_{n=1}^{N_{E}}\psi_{S_{a}E_{t}}+1},\psi_{E,2}^{\mathrm{MRC}}=a_{2}\sum_{n=1}^{N_{E}}\psi_{S_{a}E_{t}}, \end{array}$$

where the source selects the *a*-th antenna to transmit data to the destination.

**Remark** **2.** *To further decrease the number of time slots used for the transmission, the source can send more than two encoded packets to the destination at each time slot. However, when more signals are combined by the source, the implementation is more complex. Moreover, the fraction of the transmit power allocated to the signals is lower, which can degrade the system performance. For example, let us consider $\psi_{D,1}^{\mathrm{TAS}/\mathrm{SC}}$ in *(17)* which can be approximated as*

(24) $$\begin{array}{c} \psi_{D,1}^{\mathrm{TAS}/\mathrm{SC}}\approx \frac{a_{1}\max_{m=1,2,\ldots ,N_{S}}\left(\max_{n=1,2,\ldots ,N_{D}}\left(\psi_{S_{m}D_{n}}\right)\right)}{a_{2}\max_{m=1,2,\ldots ,N_{S}}\left(\max_{n=1,2,\ldots ,N_{D}}\left(\psi_{S_{m}D_{n}}\right)\right)}=\frac{a_{1}}{a_{2}}. \end{array}$$

*It is obvious from *(24)* that to obtain high SNR $\psi_{D,1}^{\mathrm{TAS}/\mathrm{SC}}$, $a_{1}$ should be much higher than $a_{2}$, (or $a_{2}$ is small). For another example, if the source combines 3 signals using the coefficients $a_{1}$, $a_{2}$ and $a_{3}$, where $a_{1}$ > $a_{2}$ > $a_{3}$ and $a_{1}+a_{2}+a_{3}=1$, similarly, we have $a_{1}$ >> $a_{2}$ >> $a_{3}$, and hence the transmit power allocated to the third signal is very small.*

**Remark** **3.** *It is obvious that to obtain the packet $p_{2}$, the destination must correctly decode the packet $p_{1}$ first. If the decoding status of $p_{1}$ is not successful, $p_{2}$ cannot also be decoded successfully. Therefore, the probabilities that in one time slot the destination cannot obtain any packet only obtains $p_{1}$, and obtains $p_{1}$ and $p_{2}$ are formulated respectively as*

(25) $$\begin{array}{cc} \chi_{D,0} & =\mathrm{Pr}\left(\psi_{D,1}^{Y}<\gamma_{\mathrm{th}}\right),\\ \chi_{D,1} & =\mathrm{Pr}\left(\psi_{D,1}^{Y}\geq \gamma_{\mathrm{th}},\psi_{D,2}^{Y}<\gamma_{\mathrm{th}}\right),\\ \chi_{D,2} & =\mathrm{Pr}\left(\psi_{D,1}^{Y}\geq \gamma_{\mathrm{th}},\psi_{D,2}^{Y}\geq \gamma_{\mathrm{th}}\right), \end{array}$$

*where $Y\in \left\{\mathrm{TAS}/\mathrm{SC},\mathrm{TAS}/\mathrm{MRC}\right\}$.*

Similarly, the probabilities that the eavesdropper cannot obtain any packet only obtains $p_{1}$, and obtains both $p_{1}$ and $p_{2}$ are formulated respectively as

(26) $$\begin{array}{cc} \chi_{E,0} & =\mathrm{Pr}\left(\psi_{E,1}^{Z}<\gamma_{\mathrm{th}}\right),\\ \chi_{E,1} & =\mathrm{Pr}\left(\psi_{E,1}^{Z}\geq \gamma_{\mathrm{th}},\psi_{E,2}^{Z}<\gamma_{\mathrm{th}}\right),\\ \chi_{E,2} & =\mathrm{Pr}\left(\psi_{E,1}^{Z}\geq \gamma_{\mathrm{th}},\psi_{E,2}^{Z}\geq \gamma_{\mathrm{th}}\right). \end{array}$$

where $Z\in \left\{\mathrm{SC},\mathrm{MRC}\right\}$.

## 3. Performance Analysis

In this section, we derive exact expressions of average number of time slots (TS) and intercept probability (IP) of the proposed protocols. At first, the probabilities $\rho_{D},\rho_{E},\chi_{D,i}$ and $\chi_{E,i}$ (i=0,1,2) are calculated.

### 3.1. Derivation of $\rho_{D}$ and $\rho_{E}$

- Case 1: The SC combiner is used by D and E

Combining (1), (7) and (13), we can obtain

(27) $$\begin{array}{cc} \rho_{D} & =1-\mathrm{Pr}\left(\max_{m=1,2,\ldots ,N_{S}}\left(\max_{n=1,2,\ldots ,N_{D}}\left(\psi_{S_{m}D_{n}}\right)\right)<\gamma_{\mathrm{th}}\right)\\ \; & =1-\prod_{m=1}^{N_{S}}\prod_{n=1}^{N_{D}}F_{\gamma_{S_{m}D_{n}}}\left(\frac{\gamma_{\mathrm{th}}}{\Delta }\right)\\ \; & =1-{\left[1-\exp\left(-\frac{\lambda_{\mathrm{SD}}\gamma_{\mathrm{th}}}{\Delta }\right)\right]}^{N_{S}N_{D}}. \end{array}$$

Similarly, combining (1), (8), and (13), the probability $\rho_{E}$ is calculated as

(28) $$\begin{array}{cc} \rho_{E} & =1-\mathrm{Pr}\left(\max_{t=1,2,\ldots ,N_{E}}\left(\psi_{S_{a}E_{t}}\right)<\gamma_{\mathrm{th}}\right)\\ \; & =1-{\left[1-\exp\left(-\frac{\lambda_{\mathrm{SE}}\gamma_{\mathrm{th}}}{\Delta }\right)\right]}^{N_{E}}. \end{array}$$

- Case 2: The MRC combiner is used by D and E

From (1), (11) and (13), the probability $\rho_{D}$ can be formulated as

(29) $$\begin{array}{cc} \rho_{D} & =1-\mathrm{Pr}\left(\max_{m=1,2,\ldots ,N_{S}}\left(\sum_{n=1}^{N_{D}}\psi_{S_{m}D_{n}}\right)<\gamma_{\mathrm{th}}\right)\\ \; & =1-{\left[\mathrm{Pr}\left(\sum_{n=1}^{N_{D}}\psi_{S_{m}D_{n}}<\gamma_{\mathrm{th}}\right)\right]}^{N_{S}}. \end{array}$$

Using CDF of sum of identical and independent exponential RVs [59], we can obtain

(30) $$\begin{array}{c} \rho_{D}=1-{\left[1-\sum_{m=0}^{N_{D}-1}\frac{1}{m!}{\left(\frac{\lambda_{\mathrm{SD}}\gamma_{\mathrm{th}}}{\Delta }\right)}^{m}\exp\left(-\frac{\lambda_{\mathrm{SD}}\gamma_{\mathrm{th}}}{\Delta }\right)\right]}^{N_{S}}. \end{array}$$

Similarly, we can calculate the probability $\rho_{E}$ in this case as follows:

(31) $$\begin{array}{c} \rho_{E}=\sum_{t=0}^{N_{E}-1}\frac{1}{t!}{\left(\frac{\lambda_{\mathrm{SE}}\gamma_{\mathrm{th}}}{\Delta }\right)}^{t}\exp\left(-\frac{\lambda_{\mathrm{SE}}\gamma_{\mathrm{th}}}{\Delta }\right). \end{array}$$

### 3.2. Derivation of $\chi_{D,i}$ and $\chi_{E,i}$

- Case 1: The SC combiner is used by D and E

At first, we consider $\chi_{D,2}$ combining (17) and (25), we have

(32) $$\begin{array}{cc} \; & \chi_{D,2}=\\ \; & \mathrm{Pr}\;\left(\;\left(a_{1}-a_{2}\gamma_{\mathrm{th}}\right)\max_{m=1,2,\ldots ,N_{S}}\;\left(\max_{n=1,2,\ldots ,N_{D}}\left(\psi_{S_{m}D_{n}}\right)\;\right)\geq \gamma_{\mathrm{th}},a_{2}\max_{m=1,2,\ldots ,N_{S}}\left(\max_{n=1,2,\ldots ,N_{D}}\left(\psi_{S_{m}D_{n}}\right)\;\right)\geq \gamma_{\mathrm{th}}\;\right)\;. \end{array}$$

We observe from (32) that if $a_{1}-a_{2}\gamma_{\mathrm{th}}\leq 0$, then $\chi_{D,2}=0$. Otherwise, (32) can be rewritten as

(33) $$\begin{array}{c} \chi_{D,2}=\mathrm{Pr}\left(\max_{m=1,2,\ldots ,N_{S}}\left(\max_{n=1,2,\ldots ,N_{D}}\left(\gamma_{S_{m}D_{n}}\right)\right)\geq \mu_{1},\max_{m=1,2,\ldots ,N_{S}}\left(\max_{n=1,2,\ldots ,N_{D}}\left(\gamma_{S_{m}D_{n}}\right)\right)\geq \mu_{2}\right), \end{array}$$

where

(34) $$\begin{array}{c} \mu_{1}=\frac{\gamma_{\mathrm{th}}}{\left(a_{1}-a_{2}\gamma_{\mathrm{th}}\right)\Delta },\mu_{2}=\frac{\gamma_{\mathrm{th}}}{a_{2}\Delta }. \end{array}$$

**Remark** **4.** *As mentioned in Remark 2, $a_{1}$ should be much higher than $a_{2}$ so that the obtained SNR $\psi_{D,1}^{\mathrm{TAS}/\mathrm{SC}}$ is high enough. Therefore, it can be assumed that $a_{1}>\left(1+\gamma_{\mathrm{th}}\right)a_{2}$, which yields the following result: $0<\mu_{1}<\mu_{2}$. Then, the probability $\chi_{D,2}$ is calculated as*

(35) $$\begin{array}{cc} \chi_{D,2} & =\mathrm{Pr}\left(\max_{m=1,2,\ldots ,N_{S}}\left(\max_{n=1,2,\ldots ,N_{D}}\left(\gamma_{S_{m}D_{n}}\right)\right)\geq \mu_{2}\right)\\ \; & =1-\mathrm{Pr}\left(\max_{m=1,2,\ldots ,N_{S}}\left(\max_{n=1,2,\ldots ,N_{D}}\left(\gamma_{S_{m}D_{n}}\right)\right)<\mu_{2}\right)\\ \; & =1-{\left[1-\exp\left(-\lambda_{\mathrm{SD}}\mu_{2}\right)\right]}^{N_{S}N_{D}}. \end{array}$$

Next, we can calculate $\chi_{D,0}$ and $\chi_{D,1}$ respectively as

(36) $$\begin{array}{cc} \chi_{D,0} & =\mathrm{Pr}\left(\max_{m=1,2,\ldots ,N_{S}}\left(\max_{n=1,2,\ldots ,N_{D}}\left(\gamma_{S_{m}D_{n}}\right)\right)<\mu_{1}\right)\\ \; & ={\left(1-\exp\left(-\lambda_{\mathrm{SD}}\mu_{1}\right)\right)}^{N_{S}N_{D}},\\ \chi_{D,1} & =\mathrm{Pr}\left(\mu_{1}\leq \max_{m=1,2,\ldots ,N_{S}}\left(\max_{n=1,2,\ldots ,N_{D}}\left(\gamma_{S_{m}D_{n}}\right)\right)<\mu_{2}\right)\\ \; & ={\left(1-\exp\left(-\lambda_{\mathrm{SD}}\mu_{2}\right)\right)}^{N_{S}N_{D}}-{\left(1-\exp\left(-\lambda_{\mathrm{SD}}\mu_{1}\right)\right)}^{N_{S}N_{D}}. \end{array}$$

Similarly, we can calculate $\chi_{E,0}$, $\chi_{E,1}$, and $\chi_{E,2}$, respectively as

(37) $$\begin{array}{cc} \chi_{E,0} & ={\left(1-\exp\left(-\lambda_{\mathrm{SE}}\mu_{1}\right)\right)}^{N_{E}},\\ \chi_{E,1} & ={\left(1-\exp\left(-\lambda_{\mathrm{SE}}\mu_{2}\right)\right)}^{N_{E}}-{\left(1-\exp\left(-\lambda_{\mathrm{SE}}\mu_{1}\right)\right)}^{N_{E}},\\ \chi_{E,2} & =1-{\left(1-\exp\left(-\lambda_{\mathrm{SE}}\mu_{2}\right)\right)}^{N_{E}}. \end{array}$$

- Case 2: The MRC combiner is used by D and E

In this case, it is straightforward to obtain the following results:

(38) $$\begin{array}{cc} \chi_{D,0} & ={\left[1-\sum_{m=0}^{N_{D}-1}\frac{{\left(\lambda_{\mathrm{SD}}\mu_{1}\right)}^{m}}{m!}\exp\left(-\lambda_{\mathrm{SD}}\mu_{1}\right)\right]}^{N_{S}},\\ \chi_{D,1} & ={\left[1-\sum_{m=0}^{N_{D}-1}\frac{{\left(\lambda_{\mathrm{SD}}\mu_{2}\right)}^{m}}{m!}\exp\left(-\lambda_{\mathrm{SD}}\mu_{2}\right)\right]}^{N_{S}}-{\left[1-\sum_{m=0}^{N_{D}-1}\frac{{\left(\lambda_{\mathrm{SD}}\mu_{1}\right)}^{m}}{m!}\exp\left(-\lambda_{\mathrm{SD}}\mu_{1}\right)\right]}^{N_{S}},\\ \chi_{D,2} & =1-{\left[1-\sum_{m=0}^{N_{D}-1}\frac{{\left(\lambda_{\mathrm{SD}}\mu_{2}\right)}^{m}}{m!}\exp\left(-\lambda_{\mathrm{SD}}\mu_{2}\right)\right]}^{N_{S}},\\ \chi_{E,0} & =1-\sum_{t=0}^{N_{E}-1}\frac{{\left(\lambda_{\mathrm{SE}}\mu_{1}\right)}^{t}}{t!}\exp\left(-\lambda_{\mathrm{SE}}\mu_{1}\right),\\ \chi_{E,1} & =\sum_{t=0}^{N_{E}-1}\frac{{\left(\lambda_{\mathrm{SE}}\mu_{1}\right)}^{t}}{t!}\exp\left(-\lambda_{\mathrm{SE}}\mu_{1}\right)-\sum_{t=0}^{N_{E}-1}\frac{{\left(\lambda_{\mathrm{SE}}\mu_{2}\right)}^{t}}{t!}\exp\left(-\lambda_{\mathrm{SE}}\mu_{2}\right),\\ \chi_{E,2} & =\sum_{t=0}^{N_{E}-1}\frac{{\left(\lambda_{\mathrm{SE}}\mu_{2}\right)}^{t}}{t!}\exp\left(-\lambda_{\mathrm{SE}}\mu_{2}\right). \end{array}$$

### 3.3. Average Number of Time Slots (TS)

#### 3.3.1. Without Using NOMA (Wo-NOMA)

The average number of time slots of the Wo-NOMA protocol can be formulated as

(39) $$\begin{array}{c} \mathrm{TS}=\sum_{N_{\mathrm{TS}}=N_{\mathrm{req}}^{\mathrm{pkt}}}^{+\infty }N_{\mathrm{TS}}\times \mathrm{Pr}\left(N_{D}^{\mathrm{pkt}}=N_{\mathrm{req}}^{\mathrm{pkt}}|N_{\mathrm{TS}}\right), \end{array}$$

where $\mathrm{Pr}\left(N_{D}^{\mathrm{pkt}}=N_{\mathrm{req}}^{\mathrm{pkt}}|N_{\mathrm{TS}}\right)$ is the probability that the destination obtains $N_{\mathrm{req}}^{\mathrm{pkt}}$ encoded packets after $N_{\mathrm{TS}}$ time slots, which follows a negative binomial distribution (see Equation (9) of [60]):

(40) $$\begin{array}{c} \mathrm{Pr}\left(N_{D}^{\mathrm{pkt}}=N_{\mathrm{req}}^{\mathrm{pkt}}|N_{\mathrm{TS}}\right)=C_{N_{\mathrm{TS}}-1}^{N_{\mathrm{req}}^{\mathrm{pkt}}-1}{\left(\rho_{D}\right)}^{N_{\mathrm{req}}^{\mathrm{pkt}}}{\left(1-\rho_{D}\right)}^{N_{\mathrm{TS}}-N_{\mathrm{req}}^{\mathrm{pkt}}}, \end{array}$$

and $C_{b}^{a}\left(b\geq a\right)$ denotes the binomial coefficient:

$$\begin{array}{c} C_{b}^{a}=\frac{b!}{a!\left(b-a\right)!}. \end{array}$$

Equation (40) can be explained as follows. After ($N_{\mathrm{TS}}-1$) time slots, the destination obtains $N_{\mathrm{req}}^{\mathrm{pkt}}-1$ encoded packets, and it correctly receives one more encoded packet at the $N_{\mathrm{TS}}$-th time slot. In (40), $C_{N_{\mathrm{TS}}-1}^{N_{\mathrm{req}}^{\mathrm{pkt}}-1}$ is number of possible cases can occur when D has $N_{\mathrm{req}}^{\mathrm{pkt}}-1$ encoded packets before the last time slot.

Substituting (40) into (39), and using Equation (8) of [60], we obtain

(41) $$\begin{array}{c} \mathrm{TS}=\frac{N_{\mathrm{req}}^{\mathrm{pkt}}}{\rho_{D}}. \end{array}$$

Substituting (27) and (29) into (41), we respectively obtain exact expressions of TS when the SC and MRC combiners are used.

#### 3.3.2. Using NOMA

In this protocol, we formulate the average number of time slots used by the source as

(42) $$\begin{array}{c} \mathrm{TS}=\sum_{N_{\mathrm{TS}}=\left\lceil N_{\mathrm{req}}^{\mathrm{pkt}}/2\right\rceil }^{+\infty }N_{\mathrm{TS}}\times \mathrm{Pr}\left(N_{D}^{\mathrm{pkt}}=N_{\mathrm{req}}^{\mathrm{pkt}}\cup N_{D}^{\mathrm{pkt}}=N_{\mathrm{req}}^{\mathrm{pkt}}+1|N_{\mathrm{TS}}\right), \end{array}$$

where $\mathrm{Pr}\left(N_{D}^{\mathrm{pkt}}=N_{\mathrm{req}}^{\mathrm{pkt}}\cup N_{D}^{\mathrm{pkt}}=N_{\mathrm{req}}^{\mathrm{pkt}}+1|N_{\mathrm{TS}}\right)$ is the probability that the destination can obtain $N_{\mathrm{req}}^{\mathrm{pkt}}$ or $N_{\mathrm{req}}^{\mathrm{pkt}}+1$ encoded packets after $N_{\mathrm{TS}}$ time slots.

Let us denote $T_{1}$ and $T_{2}$ as the number of time slots that the destination correctly receives one encoded packet and two encoded packets, respectively. Now, to calculate $\mathrm{Pr}\left(N_{D}^{\mathrm{pkt}}=N_{\mathrm{req}}^{\mathrm{pkt}}\cup N_{D}^{\mathrm{pkt}}=N_{\mathrm{req}}^{\mathrm{pkt}}+1|N_{\mathrm{TS}}\right)$, we consider three cases as follows:

- Case 1: After $N_{\mathrm{TS}}-1$ time slots, the destination obtains $N_{\mathrm{req}}^{\mathrm{pkt}}-2$ encoded packets, and at the last time slot, it obtains two encoded packets.

In this case, after the transmission is terminated, the destination has $N_{\mathrm{req}}^{\mathrm{pkt}}$ encoded packets, i.e., $N_{D}^{\mathrm{pkt}}=N_{\mathrm{req}}^{\mathrm{pkt}}$ and $T_{1}+2T_{2}=N_{\mathrm{req}}^{\mathrm{pkt}}$. Moreover, the probability of Case 1 can be calculated as follows:

(43) $$\begin{array}{c} \theta_{D,1}=\sum_{T_{2}=1}^{\left\lfloor N_{\mathrm{req}}^{\mathrm{pkt}}/2\right\rfloor }C_{N_{\mathrm{TS}}-1}^{T_{1}}C_{N_{\mathrm{TS}}-T_{1}-1}^{T_{2}-1}{\left(\chi_{D,2}\right)}^{T_{2}}{\left(\chi_{D,1}\right)}^{T_{1}}{\left(\chi_{D,0}\right)}^{N_{\mathrm{TS}}-T_{2}-T_{1}}, \end{array}$$

where $T_{1}\leq N_{\mathrm{TS}}-1$, $T_{2}\leq N_{\mathrm{TS}}-T_{1}$.

- Case 2: After $N_{\mathrm{TS}}-1$ time slots, the destination obtains $N_{\mathrm{req}}^{\mathrm{pkt}}-1$ encoded packets, and at the last time slot, it only obtains one encoded packet.

In Case 2, we also have $N_{D}^{\mathrm{pkt}}=N_{\mathrm{req}}^{\mathrm{pkt}}$ and $T_{1}+2T_{2}=N_{\mathrm{req}}^{\mathrm{pkt}}$. Then, the probability of this event is computed as

(44) $$\begin{array}{c} \theta_{D,2}=\sum_{T_{2}=0}^{\left\lfloor N_{\mathrm{req}}^{\mathrm{pkt}}/2\right\rfloor }C_{N_{\mathrm{TS}}-1}^{T_{2}}C_{N_{\mathrm{TS}}-T_{2}-1}^{T_{1}-1}{\left(\chi_{D,2}\right)}^{T_{2}}{\left(\chi_{D,1}\right)}^{T_{1}}{\left(\chi_{D,0}\right)}^{N_{\mathrm{TS}}-T_{2}-T_{1}}, \end{array}$$

where $1\leq T_{1}\leq N_{\mathrm{TS}}-T_{2}$.

- Case 3: After $N_{\mathrm{TS}}-1$ time slots, the destination obtains $N_{\mathrm{req}}^{\mathrm{pkt}}-1$ encoded packets, and at the last time slot, it obtains two encoded packets.

In this case, the destination can successfully receive $N_{\mathrm{req}}^{\mathrm{pkt}}+1$ encoded packets after $N_{\mathrm{TS}}$ time slots: $T_{1}+2T_{2}=N_{D}^{\mathrm{pkt}}=N_{\mathrm{req}}^{\mathrm{pkt}}+1$. Therefore, the probability that this event occurs can be calculated exactly as

(45) $$\begin{array}{c} \theta_{D,3}=\sum_{T_{2}=1}^{\left\lceil N_{\mathrm{req}}^{\mathrm{pkt}}/2\right\rceil }C_{N_{\mathrm{TS}}-1}^{T_{1}}C_{N_{\mathrm{TS}}-T_{1}-1}^{T_{2}-1}{\left(\chi_{D,2}\right)}^{T_{2}}{\left(\chi_{D,1}\right)}^{T_{1}}{\left(\chi_{D,0}\right)}^{N_{\mathrm{TS}}-T_{2}-T_{1}}, \end{array}$$

where $T_{1}\leq N_{\mathrm{TS}}-1$, $T_{2}\leq N_{\mathrm{TS}}-T_{1}$.

From (43)–(45), we can obtain an exact expression of $\mathrm{Pr}\left(N_{D}^{\mathrm{pkt}}=N_{\mathrm{req}}^{\mathrm{pkt}}\cup N_{D}^{\mathrm{pkt}}=N_{\mathrm{req}}^{\mathrm{pkt}}+1|N_{\mathrm{TS}}\right)$ by using the following formula:

$$\begin{array}{c} \mathrm{Pr}\left(N_{D}^{\mathrm{pkt}}=N_{\mathrm{req}}^{\mathrm{pkt}}\cup N_{D}^{\mathrm{pkt}}=N_{\mathrm{req}}^{\mathrm{pkt}}+1|N_{\mathrm{TS}}\right)=\theta_{D,1}+\theta_{D,2}+\theta_{D,3}. \end{array}$$

Then, from (42), we can write the average number of time slots used in the NOMA protocol as follows:

(46) $$\begin{array}{c} \mathrm{TS}=\sum_{N_{\mathrm{TS}}=\left\lceil N_{\mathrm{req}}^{\mathrm{pkt}}/2\right\rceil }^{+\infty }N_{\mathrm{TS}}\times \left(\theta_{D,1}+\theta_{D,2}+\theta_{D,3}\right). \end{array}$$

**Remark** **5.** *From *(41)* and *(46)*, we can observe that when the transmit SNR is high enough, i.e., Δ→+∞, the values of TS in the Wo-NOMA and NOMA protocols converge to $N_{\mathrm{req}}^{\mathrm{pkt}}$ and $\left\lceil N_{\mathrm{req}}^{\mathrm{pkt}}/2\right\rceil$, respectively. It is due to the fact that at high *Δ* regimes, all of the encoded packet(s) can be correctly received by the destination. Therefore, by using NOMA, the proposed protocol can reduce a half of time slots used for transmitting the encoded packets.*

### 3.4. Intercept Probability (IP)

In this subsection, we calculate the intercept probability of the proposed protocols with and without using NOMA.

#### 3.4.1. Without Using NOMA (Wo-NOMA)

At first, we see that the source data is intercepted if the eavesdropper can sufficiently obtain the number of the encoded packets for recovering the original data before or at the same time with the destination. Mathematically speaking, we can write

(47) $$\begin{array}{c} \mathrm{IP}=\sum_{N_{\mathrm{TS}}^{E}=N_{\mathrm{req}}^{\mathrm{pkt}}}^{+\infty }\left[\begin{array}{c} \left(\mathrm{Pr}\left(N_{D}^{\mathrm{pkt}}=N_{\mathrm{req}}^{\mathrm{pkt}}|N_{\mathrm{TS}}^{E}\right)+\mathrm{Pr}\left(N_{D}^{\mathrm{pkt}}<N_{\mathrm{req}}^{\mathrm{pkt}}|N_{\mathrm{TS}}^{E}\right)\right)\\ \times \mathrm{Pr}\left(N_{E}^{\mathrm{pkt}}=N_{\mathrm{req}}^{\mathrm{pkt}}|N_{\mathrm{TS}}^{E}\right) \end{array}\right], \end{array}$$

Equation (47) implies that the eavesdropper can obtain $N_{\mathrm{req}}^{\mathrm{pkt}}$ encoded packets after $N_{\mathrm{TS}}^{E}$ time slots, while the destination can sufficiently receive or not. In (47), $\mathrm{Pr}\left(N_{D}^{\mathrm{pkt}}=N_{\mathrm{req}}^{\mathrm{pkt}}|N_{\mathrm{TS}}^{E}\right)$ is calculated as in (40), and similarly, $\mathrm{Pr}\left(N_{D}^{\mathrm{pkt}}<N_{\mathrm{req}}^{\mathrm{pkt}}|N_{\mathrm{TS}}^{E}\right)$ is also given as

(48) $$\begin{array}{c} \mathrm{Pr}\left(N_{E}^{\mathrm{pkt}}=N_{\mathrm{req}}^{\mathrm{pkt}}|N_{\mathrm{TS}}^{E}\right)=C_{N_{\mathrm{TS}}^{E}-1}^{N_{\mathrm{req}}^{\mathrm{pkt}}-1}{\left(\rho_{E}\right)}^{N_{\mathrm{req}}^{\mathrm{pkt}}}{\left(1-\rho_{E}\right)}^{N_{\mathrm{TS}}^{E}-N_{\mathrm{req}}^{\mathrm{pkt}}}. \end{array}$$

Considering $\mathrm{Pr}\left(N_{D}^{\mathrm{pkt}}<N_{\mathrm{req}}^{\mathrm{pkt}}|N_{\mathrm{TS}}^{E}\right)$ in (47); this is the probability that the destination cannot sufficiently receive the number of the encoded packets for the data recovery after $N_{\mathrm{TS}}^{E}$ time slots and is calculated as

(49) $$\begin{array}{c} \mathrm{Pr}\left(N_{D}^{\mathrm{pkt}}<N_{\mathrm{req}}^{\mathrm{pkt}}|N_{\mathrm{TS}}^{E}\right)=\sum_{N_{D}^{\mathrm{pkt}}=0}^{N_{\mathrm{req}}^{\mathrm{pkt}}-1}C_{N_{\mathrm{TS}}^{E}}^{N_{D}^{\mathrm{pkt}}}{\left(\rho_{D}\right)}^{N_{D}^{\mathrm{pkt}}}{\left(1-\rho_{D}\right)}^{N_{\mathrm{TS}}^{E}-N_{D}^{\mathrm{pkt}}}. \end{array}$$

**Remark** **6.** *When the eavesdropper obtains $N_{\mathrm{req}}^{\mathrm{pkt}}$ encoded packets, it does not decode the encoded packets any more, regardless of whether the source still transmits the encoded packets to the destination. This also means that after having $N_{\mathrm{req}}^{\mathrm{pkt}}$ encoded packets, it stops overhearing the data transmission and starts the data recovery.*

Combining (47)–(49), IP can be exactly calculated as

(50) $$\begin{array}{c} \mathrm{IP}=\;\sum_{N_{\mathrm{TS}}^{E}=N_{\mathrm{req}}^{\mathrm{pkt}}}^{+\infty }\;\;\left[\begin{array}{c} \;\;\left(C_{N_{\mathrm{TS}}^{E}-1}^{N_{\mathrm{req}}^{\mathrm{pkt}}-1}{\left(\rho_{D}\right)}^{N_{\mathrm{req}}^{\mathrm{pkt}}}{\left(1-\rho_{D}\right)}^{N_{\mathrm{TS}}^{E}-N_{\mathrm{req}}^{\mathrm{pkt}}}+\sum_{N_{D}^{\mathrm{pkt}}=0}^{N_{\mathrm{req}}^{\mathrm{pkt}}-1}C_{N_{\mathrm{TS}}^{E}}^{N_{D}^{\mathrm{pkt}}}{\left(\rho_{D}\right)}^{N_{D}^{\mathrm{pkt}}}{\left(1-\rho_{D}\right)}^{N_{\mathrm{TS}}^{E}-N_{D}^{\mathrm{pkt}}}\right)\;\;\\ \;\;\times C_{N_{\mathrm{TS}}^{E}-1}^{N_{\mathrm{req}}^{\mathrm{pkt}}-1}{\left(\rho_{E}\right)}^{N_{\mathrm{req}}^{\mathrm{pkt}}}{\left(1-\rho_{E}\right)}^{N_{\mathrm{TS}}^{E}-N_{\mathrm{req}}^{\mathrm{pkt}}} \end{array}\right]. \end{array}$$

#### 3.4.2. Using NOMA

In this protocol, IP can be formulated as

(51) $$\begin{array}{c} \mathrm{IP}=\sum_{N_{\mathrm{TS}}^{E}=\left\lceil N_{\mathrm{req}}^{\mathrm{pkt}}/2\right\rceil }^{+\infty }\left[\begin{array}{c} \left(\mathrm{Pr}\left(N_{D}^{\mathrm{pkt}}=N_{\mathrm{req}}^{\mathrm{pkt}}\cup N_{D}^{\mathrm{pkt}}=N_{\mathrm{req}}^{\mathrm{pkt}}+1|N_{\mathrm{TS}}^{E}\right)+\mathrm{Pr}\left(N_{D}^{\mathrm{pkt}}<N_{\mathrm{req}}^{\mathrm{pkt}}|N_{\mathrm{TS}}^{E}\right)\right)\\ \times \mathrm{Pr}\left(N_{E}^{\mathrm{pkt}}=N_{\mathrm{req}}^{\mathrm{pkt}}\cup N_{E}^{\mathrm{pkt}}=N_{\mathrm{req}}^{\mathrm{pkt}}+1|N_{\mathrm{TS}}^{E}\right) \end{array}\right]. \end{array}$$

where $\mathrm{Pr}\left(N_{D}^{\mathrm{pkt}}=N_{\mathrm{req}}^{\mathrm{pkt}}\cup N_{D}^{\mathrm{pkt}}=N_{\mathrm{req}}^{\mathrm{pkt}}+1|N_{\mathrm{TS}}^{E}\right)$ and $\mathrm{Pr}\left(N_{E}^{\mathrm{pkt}}=N_{\mathrm{req}}^{\mathrm{pkt}}\cup N_{E}^{\mathrm{pkt}}=N_{\mathrm{req}}^{\mathrm{pkt}}+1|N_{\mathrm{TS}}^{E}\right)$ are computed similarly to (43)–(45) as

(52) $$\begin{array}{cc} \mathrm{Pr}\left(N_{D}^{\mathrm{pkt}}=N_{\mathrm{req}}^{\mathrm{pkt}}\cup N_{D}^{\mathrm{pkt}}=N_{\mathrm{req}}^{\mathrm{pkt}}+1|N_{\mathrm{TS}}^{E}\right) & =\theta_{D,1}+\theta_{D,2}+\theta_{D,3},\\ \mathrm{Pr}\left(N_{E}^{\mathrm{pkt}}=N_{\mathrm{req}}^{\mathrm{pkt}}\cup N_{E}^{\mathrm{pkt}}=N_{\mathrm{req}}^{\mathrm{pkt}}+1|N_{\mathrm{TS}}^{E}\right) & =\theta_{E,1}+\theta_{E,2}+\theta_{E,3}. \end{array}$$

In (52), we note that $\theta_{D,1}$, $\theta_{D,2}$, and $\theta_{D,3}$ are obtained by replacing $N_{\mathrm{TS}}$ in (43)–(45) by $N_{\mathrm{TS}}^{E}$. For $\theta_{E,1}$, $\theta_{E,2}$, and $\theta_{E,3}$, with the same method as deriving $\theta_{D,1}$, $\theta_{D,2}$, $\theta_{D,3}$, we can obtain

(53) $$\begin{array}{cc} \theta_{E,1} & =\sum_{V_{2}=1}^{\left\lfloor N_{\mathrm{req}}^{\mathrm{pkt}}/2\right\rfloor }C_{N_{\mathrm{TS}}^{E}-1}^{V_{1}}C_{N_{\mathrm{TS}}^{E}-V_{1}-1}^{V_{2}-1}{\left(\chi_{E,2}\right)}^{V_{2}}{\left(\chi_{E,1}\right)}^{V_{1}}{\left(\chi_{E,0}\right)}^{N_{\mathrm{TS}}^{E}-V_{2}-V_{1}},\\ \theta_{E,2} & =\sum_{V_{2}=0}^{\left\lfloor N_{\mathrm{req}}^{\mathrm{pkt}}/2\right\rfloor }C_{N_{\mathrm{TS}}^{E}-1}^{V_{2}}C_{N_{\mathrm{TS}}^{E}-V_{2}-1}^{V_{1}-1}{\left(\chi_{E,2}\right)}^{V_{2}}{\left(\chi_{E,1}\right)}^{V_{1}}{\left(\chi_{E,0}\right)}^{N_{\mathrm{TS}}^{E}-V_{2}-V_{1}},\\ \theta_{E,3} & =\sum_{V_{2}=1}^{\left\lceil N_{\mathrm{req}}^{\mathrm{pkt}}/2\right\rceil }C_{N_{\mathrm{TS}}^{E}-1}^{V_{1}}C_{N_{\mathrm{TS}}^{E}-V_{1}-1}^{V_{2}-1}{\left(\chi_{E,2}\right)}^{V_{2}}{\left(\chi_{E,1}\right)}^{V_{1}}{\left(\chi_{E,0}\right)}^{N_{\mathrm{TS}}^{E}-V_{2}-V_{1}}, \end{array}$$

where $V_{1}$ and $V_{2}$ are the number of time slots that the eavesdropper correctly receives one encoded packet and two encoded packets, respectively.

Considering $\mathrm{Pr}\left(N_{D}^{\mathrm{pkt}}<N_{\mathrm{req}}^{\mathrm{pkt}}|N_{\mathrm{TS}}^{E}\right)$; this is probability that the destination cannot obtain $N_{\mathrm{req}}^{\mathrm{pkt}}$ encoded packets after $N_{\mathrm{TS}}^{E}$ time slots, and is computed as

(54) $$\begin{array}{cc} \mathrm{Pr}\left(N_{D}^{\mathrm{pkt}}<N_{\mathrm{req}}^{\mathrm{pkt}}|N_{\mathrm{TS}}^{E}\right) & \overset{\Delta }{\;=}\theta_{D,4}\\ \; & =\sum_{N_{D}^{\mathrm{pkt}}=0}^{N_{\mathrm{req}}^{\mathrm{pkt}}-1}\sum_{T_{2}=0}^{\left\lfloor N_{D}^{\mathrm{pkt}}/2\right\rfloor }C_{N_{\mathrm{TS}}^{E}}^{T_{2}}C_{N_{\mathrm{TS}}^{E}-T_{2}}^{T_{1}}{\left(\chi_{D,2}\right)}^{T_{2}}{\left(\chi_{D,1}\right)}^{T_{1}}{\left(\chi_{D,0}\right)}^{N_{\mathrm{TS}}^{E}-T_{2}-T_{1}}. \end{array}$$

From (51)–(54), IP in the NOMA protocol is written as follows:

(55) $$\begin{array}{c} \mathrm{IP}=\sum_{N_{\mathrm{TS}}^{E}=\left\lceil N_{\mathrm{req}}^{\mathrm{pkt}}/2\right\rceil }^{+\infty }\left[\left(\theta_{D,1}+\theta_{D,2}+\theta_{D,3}+\theta_{D,4}\right)\times \left(\theta_{E,1}+\theta_{E,2}+\theta_{E,3}\right)\right]. \end{array}$$

**Remark** **7.** *Equations *(50)* and *(55)* exactly express the IP performance of the Wo-NOMA and NOMA protocols. To obtain the IP values, we truncate the infinite series by 500 first terms. Moreover, because *(50)* and *(55)* are in closed-form formulas, which can be used efficiently in designing and optimizing the networks.*

## 4. Simulation Results

In this section, we present simulation results using the Monte-Carlo approach to verify the theoretical results obtained in Section 3 as well as to compare the performances of the proposed protocols with and without using NOMA, in terms of TS and IP. All of the simulation and theoretical results are drawn by MATLAB R2014a software (MathWorks, Natick, MA, USA). For Monte-Carlo simulations, we perform $10^{5}-5\times 10^{6}$ trials in which the Rayleigh channel coefficients of the X-Y links are generated by hXY=1/sqrt(2×LXY)×(randn(1,1)+j×randn(1,1)), where $\left(X,Y\right)\in \left\{S_{m},D_{n},E_{t}\right\}$, LXY (or $\lambda_{\mathrm{XY}}$) is the parameter of the X-Y channel, and randn(1,1) is a MATLAB function which generates Gaussian distributed pseudo-random numbers with zero-mean and unit variance. Then, using the given system parameters (we summarize the system parameters and their value ranges in Table 1), we obtain the simulation results of TS and IP. For the theoretical results, the expressions of TS and IP derived in the previous section are used to present them. As mentioned in Remark 7, the infinite series in the derived formulas are truncated by 500 first terms.

### 4.1. Average Number of Time Slots (TS)

**Figure 2:**
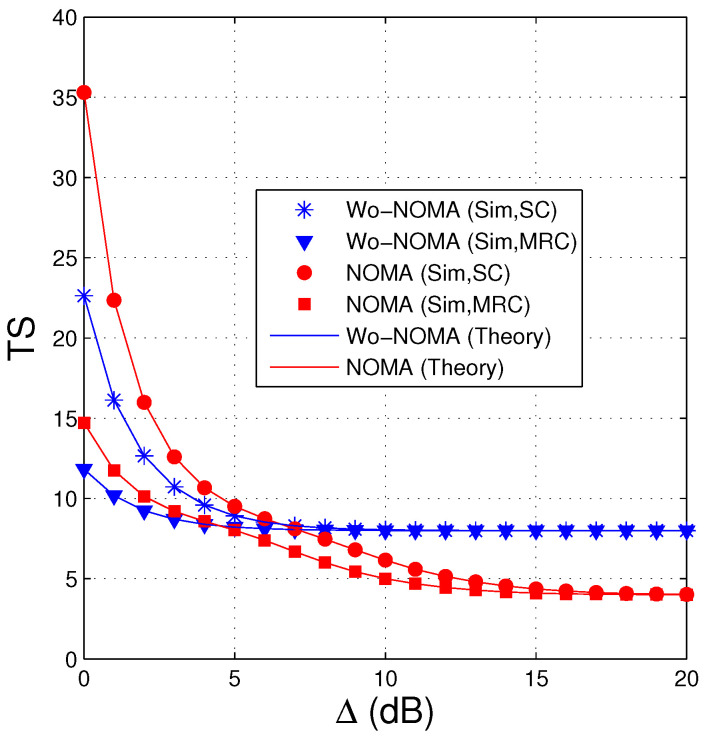
Average number of time slots as a function of Δ (dB) when $N_{S}=1$, $N_{D}=3$, $\lambda_{\mathrm{SD}}=2$, $N_{\mathrm{req}}^{\mathrm{pkt}}=8$, $a_{1}=0.9$, $\gamma_{\mathrm{th}}=1$.

In Figure 2, we present average number of time slots that the source uses to transmit the encoded packets to the destination as a function of the transmit SNR (Δ) in dB. In this figure, the number of antennas at the source ($N_{S}$) and the destination ($N_{D}$) is 1 and 3, respectively, the parameter of the data link ($\lambda_{\mathrm{SD}}$) is fixed by 2, the required number of the encoded packets for successfully recovering the original data ($N_{\mathrm{req}}^{\mathrm{pkt}}$) is set by 8, and the threshold $\gamma_{\mathrm{th}}$ is set to 1. As mentioned in Remark 4, the value of $a_{1}$ has to satisfy the condition: $a_{1}>\left(1+\gamma_{\mathrm{th}}\right)a_{2}$ or $a_{1}>\left(1+\gamma_{\mathrm{th}}\right)/\left(2+\gamma_{\mathrm{th}}\right)=2/3$, hence we can select $a_{1}=0.9$ ($a_{2}=0.1$). We can see from Figure 2 that the TS values of the Wo-NOMA and NOMA protocols decrease with the increasing of Δ and are lower when the destination is equipped with the MRC combiner. However, at high Δ regions, the TS values of the Wo-NOMA protocol converge to $N_{\mathrm{req}}^{\mathrm{pkt}}$, while those of the NOMA protocol reach to $N_{\mathrm{req}}^{\mathrm{pkt}}/2$. It is due to the fact that at high transmit SNR, the destination in the NOMA scheme can obtain two encoded packets at each time slot, and hence the source only uses $N_{\mathrm{req}}^{\mathrm{pkt}}/2$ time slots for the data transmission. However, we can observe that the NOMA protocol does not perform well at low Δ values when it uses more time slots than the Wo-NOMA protocol. It is worth noting that the simulation results (Sim) match very well with the theoretical ones (Theory), which verifies our derivations.

**Figure 3:**
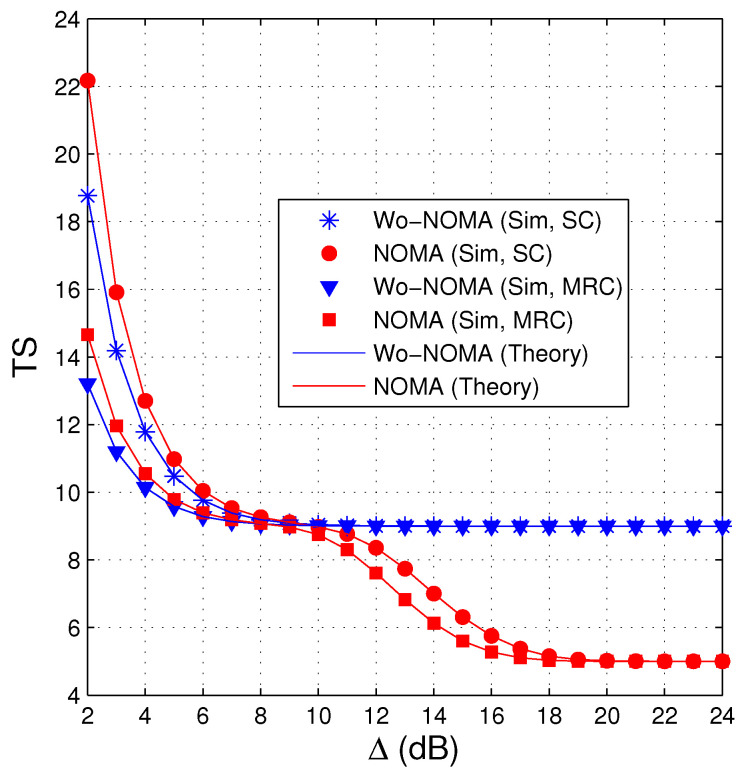
Average number of time slots as a function of Δ (dB) when $N_{S}=2$, $N_{D}=2$, $\lambda_{\mathrm{SD}}=3$, $N_{\mathrm{req}}^{\mathrm{pkt}}=9$, $a_{1}=0.95$, $\gamma_{\mathrm{th}}=1$.

Figure 3 shows similar results to Figure 2, i.e., the performance of the NOMA protocol is better than that of the Wo-NOMA protocol at medium and high transmit SNRs. We also see from Figure 3 that the TS values of Wo-NOMA and NOMA at high Δ regimes converge to $N_{\mathrm{req}}^{\mathrm{pkt}}$ and $\left\lceil N_{\mathrm{req}}^{\mathrm{pkt}}/2\right\rceil$, respectively. In addition, as shown in Figure 2 and Figure 3, the TS performance of Wo-NOMA more rapidly converges than that of NOMA. Again, the simulation results validate the correction of the theoretical ones.

**Figure 4:**
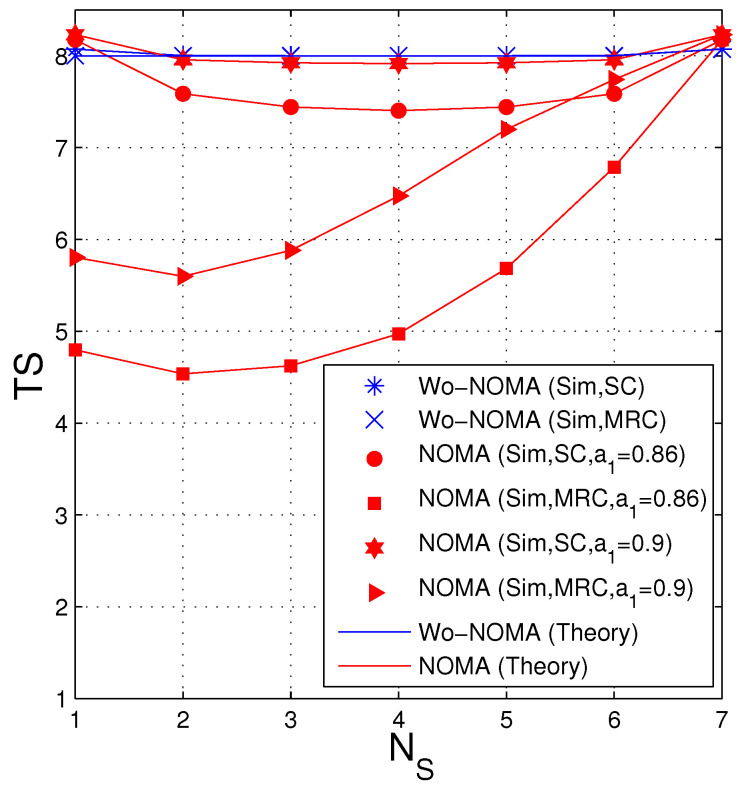
Average number of time slots as a function of $N_{S}$ when Δ=8 dB, $N_{D}+N_{S}=8$, $\lambda_{\mathrm{SD}}=3$, $N_{\mathrm{req}}^{\mathrm{pkt}}=8$, $\gamma_{\mathrm{th}}=1.5$.

In Figure 4, we fix the total number of antennas at the source and the destination, i.e., $N_{S}+N_{D}=8$, and present TS as a function of $N_{S}$. In this figure, the Wo-NOMA protocol almost uses 8 time slots for transmitting the encoded packets, for all $N_{S}$. In the NOMA protocol, the average number of time slots significantly varies as changing $N_{S}$ from 1 to 7. We can see that with the SC technique, the TS performance of the NOMA protocol is same when the number of antennas at the source is $N_{S}$ and $8-N_{S}$. Moreover, in this case, the value of TS is lowest when $N_{S}=N_{D}=4$. However, in the case where the destination is equipped with MRC, the optimal value of $N_{S}$ is 2 ($N_{D}=6$), and the TS performance is worst as $N_{S}=7$. It is due to the fact that the MRC combiner is better than the SC one, and hence more antennas should be allocated to the destination to optimize the TS performance. Finally, it is seen that the TS values of the NOMA scheme with $a_{1}=0.86$ ($a_{2}=0.14$) are lower than those with $a_{1}=0.9$ ($a_{2}=0.1$). This can be explained as follows: Reducing $a_{2}$ means that the transmit power of the second signal is lower, which hence decreases the probability that the destination can obtain two encoded packets in each time slot (see $\chi_{D,2}$ in (35) and (38)), as well as increases the average number of the time slots used.

### 4.2. Intercept Probability (IP)

**Figure 5:**
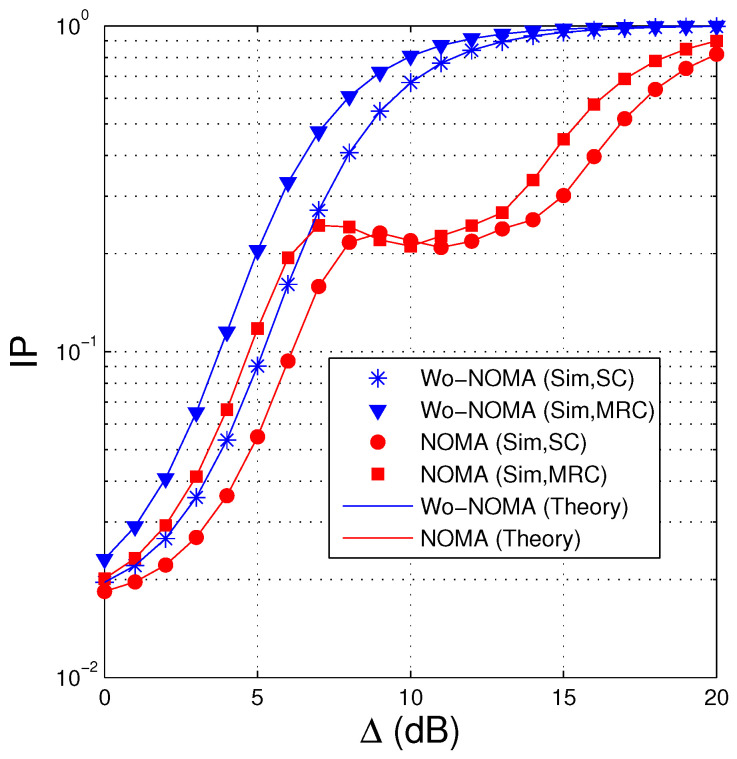
Intercept probability as a function of Δ (dB) when $N_{S}=3$, $N_{D}=2$, $N_{E}=2$, $\lambda_{\mathrm{SD}}=2.5$, $\lambda_{\mathrm{SE}}=2.5$, $N_{\mathrm{req}}^{\mathrm{pkt}}=8$, $a_{1}=0.9$, $\gamma_{\mathrm{th}}=1$.

In Figure 5, we present IP of the proposed protocols as a function of Δ in dB. We can see that IP of the Wo-NOMA and NOMA protocols almost increases as increasing the transmit SNR. It is due to the fact that when the transmit power of the source is high, SNR of the eavesdropping link also increases, which enhances the intercept probability. However, in the NOMA scheme, when Δ belongs to interval of (8 dB, 10 dB), IP slightly decreases with the increasing of Δ, and hence, there exists a high performance gap between Wo-NOMA and NOMA in this interval. Because the intercept probability at the eavesdropper depends on the decoding at the destination and the interference between the signals, the changing of IP in the NOMA protocol, with respect to Δ, is more complex. Indeed, from (17), (18), (22) and (23), it is observed that as Δ increases, the interference from $x_{2}\left[u\right]$ to $x_{1}\left[u\right]$ also increases, which leads to a slow increase of SNR of $x_{1}\left[u\right]$ obtained at the D and E nodes. Because D and E must decode $x_{1}\left[u\right]$ first, the slow increase of SNRs can make IP slightly increase. However, when Δ is high enough, all the encoded packets can be correctly obtained by D and E. In this case, D and E can obtain $N_{\mathrm{req}}^{\mathrm{pkt}}$ encoded packets at the same time, and hence the IP value converges to 1, as shown in Figure 5. Next, we can observe that when the destination and the eavesdropper use MRC, the IP values of the proposed protocols are higher. It is because the intercept possibility of the eavesdropper is better when it is equipped with MRC. Finally, it is seen that the NOMA protocol obtains better IP performance compared with the Wo-NOMA one.

**Figure 6:**
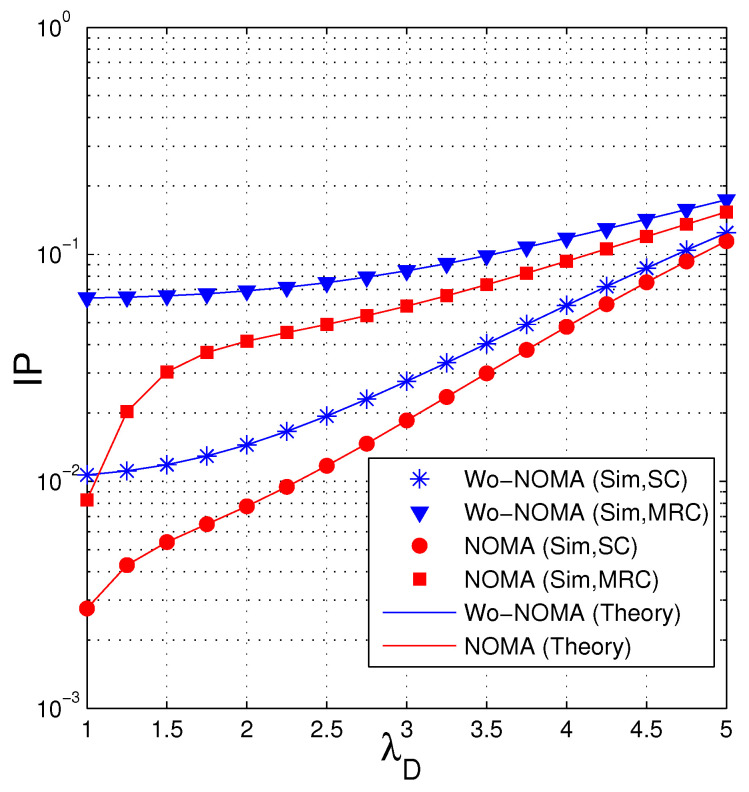
Intercept probability as a function of $\lambda_{\mathrm{SD}}$ when Δ=7 (dB), $N_{S}=2$, $N_{D}=2$, $N_{E}=2$, $\lambda_{\mathrm{SE}}=5$, $N_{\mathrm{req}}^{\mathrm{pkt}}=9$, $a_{1}=0.95$, $\gamma_{\mathrm{th}}=1$.

In Figure 6, we investigate the impact of the parameter of the data link ($\lambda_{\mathrm{SD}}$) on the IP performance. As we can see, IP of the proposed protocols increases as $\lambda_{\mathrm{SD}}$ increases. It is due to the fact that when the quality of the data channel is worse ($\lambda_{\mathrm{SD}}$ is high), the eavesdropper has more opportunity to obtain sufficient number of the encoded packets for recovering the original data. We also see that the IP performance of the Wo-NOMA protocol is worse than that of the NOMA protocol. Similar to Figure 5, the intercept probability of the eavesdropper increases when it uses MRC.

**Figure 7:**
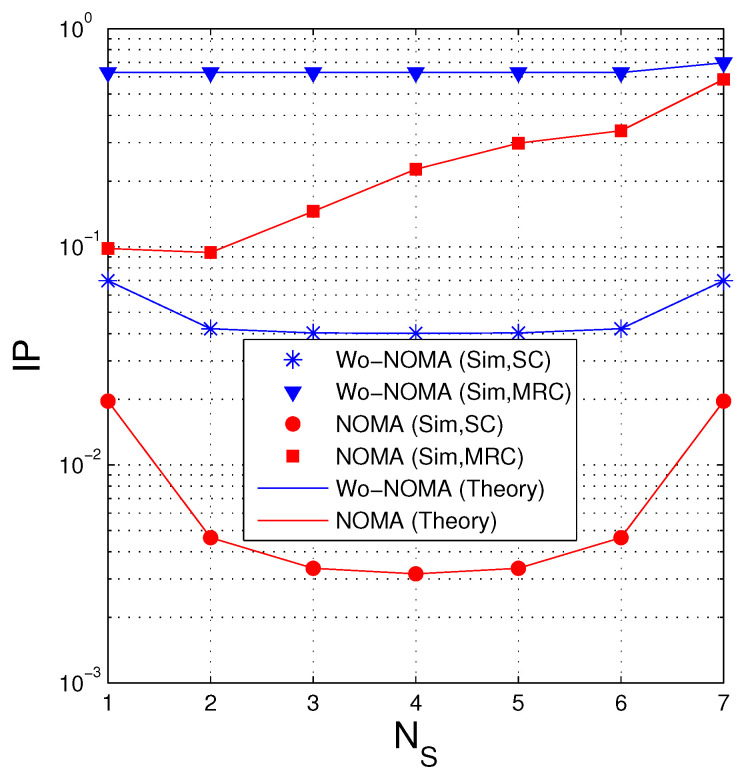
Intercept probability as a function of $N_{S}$ when Δ=5 (dB), $N_{S}+N_{D}=8$, $N_{E}=4$, $\lambda_{\mathrm{SD}}=2$, $\lambda_{\mathrm{SE}}=3$, $N_{\mathrm{req}}^{\mathrm{pkt}}=8$, $a_{1}=0.9$, $\gamma_{\mathrm{th}}=1.5$.

**Figure 8:**
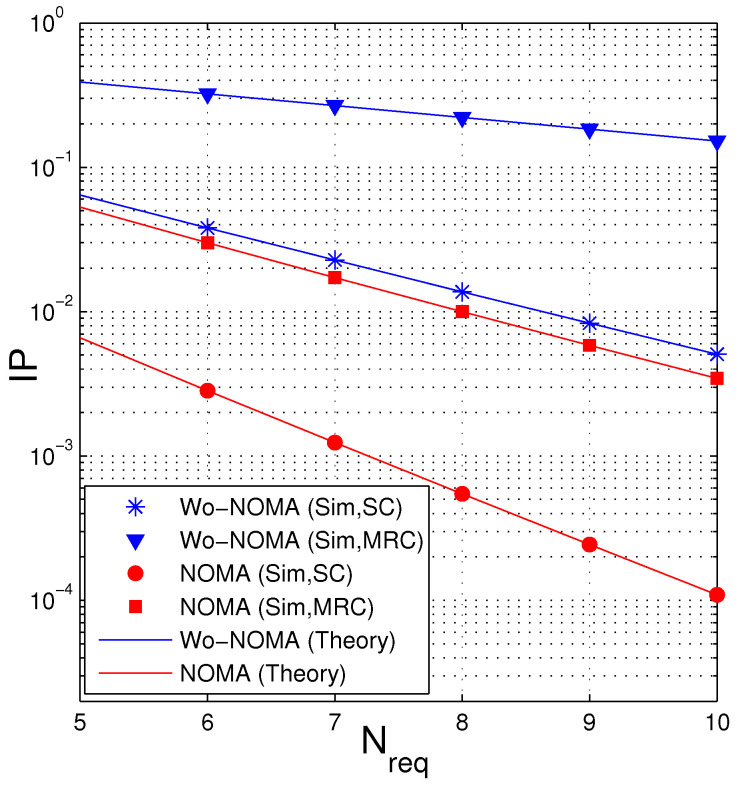
Intercept probability as a function of $N_{\mathrm{req}}^{\mathrm{pkt}}$ when Δ=5 (dB), $N_{S}=3$, $N_{D}=3$, $N_{E}=3$, $\lambda_{\mathrm{SD}}=2$, $\lambda_{\mathrm{SE}}=3$, $a_{1}=0.85$, $\gamma_{\mathrm{th}}=1.5$.

**Figure 9:**
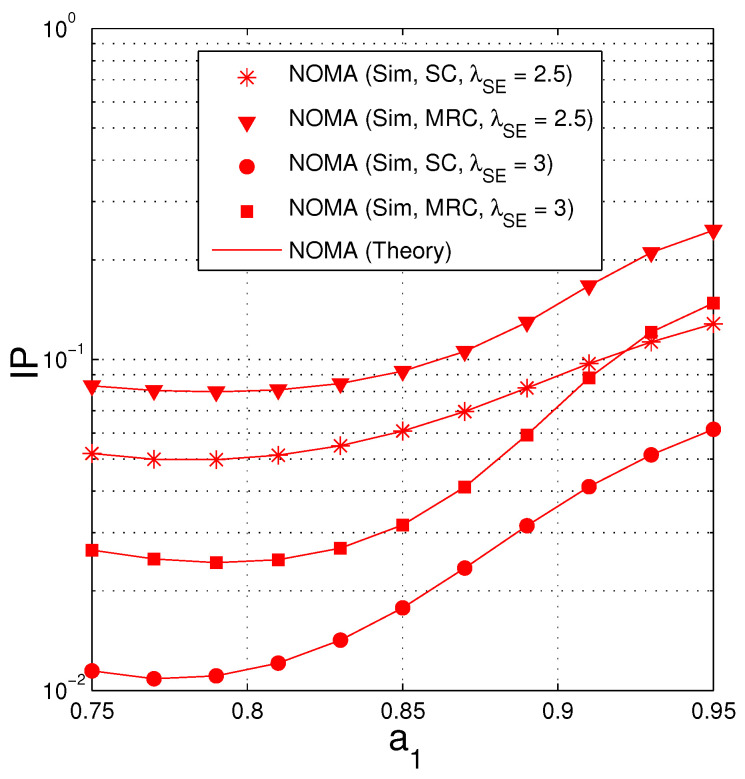
Intercept probability as a function of $a_{1}$ when Δ=7.5 (dB), $N_{S}=2$, $N_{D}=2$, $N_{E}=2$, $\lambda_{\mathrm{SD}}=2$, $N_{\mathrm{req}}^{\mathrm{pkt}}=8$, $\gamma_{\mathrm{th}}=1.5$.

Figure 7 presents IP as a function of $N_{S}$ when $N_{S}+N_{D}=8$. Similar to Figure 4, in the case where the D and E nodes use SC, the IP value is lowest when $N_{S}=N_{D}=4$, and when MRC is employed, the optimal value of $N_{S}$ is 2. It is also seen that the IP performance of the Wo-NOMA protocol slightly varies with the changing of $N_{S}$ but that of the NOMA protocol significantly varies. Again, the NOMA protocol obtains better performance compared with the Wo-NOMA one. Moreover, we can see from this figure that when the MRC technique is used by the eavesdropper, the IP performance is not good. Indeed, if the desired value of IP is (below) 0.1, it is seen that both Wo-NOMA and NOMA cannot be practically implemented. In this case, to reduce IP, the source can reduce its transmit power or appropriately design the systems parameters $N_{\mathrm{req}}^{\mathrm{pkt}}$ and $a_{1}$ (see Figure 8 and Figure 9 below).

In Figure 8, we present IP of the proposed protocols as a function of $N_{\mathrm{req}}^{\mathrm{pkt}}$. For ease of observation, we only change $N_{\mathrm{req}}^{\mathrm{pkt}}$ from 5 to 10. We can see that the values of IP decrease as $N_{\mathrm{req}}^{\mathrm{pkt}}$ increases. It is due to the fact that as $N_{\mathrm{req}}^{\mathrm{pkt}}$ is higher, the probability that the destination can obtain $N_{\mathrm{req}}^{\mathrm{pkt}}$ encoded packets before the eavesdropper increases, which hence reduces the intercept probability at the eavesdropper. The obtained results in this figure can be used to design the considered network. For example, we assume that the D and E nodes are equipped with the MRC combiner, and hence Wo-NOMA cannot be used due to high IP value (higher than 0.1). Instead of Wo-NOMA, the NOMA scheme can be used to obtain higher security for the source data. For another example, assume that the system cannot use NOMA due to limited hardware and processing capacity. In this case, the source in the Wo-NOMA protocol can increase the number of $N_{\mathrm{req}}^{\mathrm{pkt}}$ to reduce IP. However, we note that increasing $N_{\mathrm{req}}^{\mathrm{pkt}}$ does increase the number of time slots and the delay time.

Figure 9 investigates the impact of the fractions of the transmit power ($a_{1}$, $a_{2}$) on the IP performance of the NOMA protocol by changing $a_{1}$, and presenting IP as a function of $a_{1}$. Again, from Remark 4, the value of $a_{1}$ must be designed so that $a_{1}>\left(1+\gamma_{\mathrm{th}}\right)/\left(2+\gamma_{\mathrm{th}}\right)=0.7143$. Hence, in this figure, we can select the interval of $a_{1}$ as $\left(0.75,0.95\right)$. As we can see, there exist optimal values of $a_{1}$ at which the IP value is lowest. It is also observed that the IP values are higher as the $\lambda_{\mathrm{SE}}$ decreases because the average channel gain of the eavesdropping is higher.

It is worth noting from Figure 4, Figure 5, Figure 6, Figure 7, Figure 8 and Figure 9 that the simulation and theoretical results are in a good agreement, which validates the derived formulas of IP.

## 5. Conclusions

This paper showed that applying the NOMA technique into FCs secure communication protocols not only reduces the number of time slots used but also enhances security. Particularly, the NOMA protocol can reduce by half the number of time slots compared with the Wo-NOMA one. For the secure transmission, IP of the eavesdropper significantly decreases as the source uses NOMA to transmit two encoded packets to the destination at each time slot. For performance illustration, we derived exact expressions of TS and IP, which were validated by computer simulations. The results showed that the performance for the Wo-NOMA and NOMA protocols can be significantly enhanced by increasing or optimally designing the number of antennas at the source and the destination, appropriately selecting the faction of transmit power allocated to the NOMA signals and increasing the number of the encoded packets required for the data recovery.

## Figures and Tables

**Table 1:** System parameters.

System Parameters	Values
Δ	0 (dB)–24 (dB)
$N_{S}$, $N_{D}$	1–7
$N_{E}$	2–4
$\lambda_{\mathrm{SD}}$	1–5
$\lambda_{\mathrm{SE}}$	2.5–5
$N_{\mathrm{req}}^{\mathrm{pkt}}$	5–10
$\gamma_{\mathrm{th}}$	1 and 1.5
$a_{1}$, $a_{2}$	$\frac{1+\gamma_{\mathrm{th}}}{2+\gamma_{\mathrm{th}}}<a_{1}<1$, $a_{2}=1-a_{1}$
